# Divergent habitat filtering of root and soil fungal communities in temperate beech forests

**DOI:** 10.1038/srep31439

**Published:** 2016-08-11

**Authors:** Kezia Goldmann, Kristina Schröter, Rodica Pena, Ingo Schöning, Marion Schrumpf, François Buscot, Andrea Polle, Tesfaye Wubet

**Affiliations:** 1 UFZ-Helmholtz-Centre for Environmental Research, Department of Soil Ecology, Theodor-Lieser-Straße 4, 06120 Halle (Saale), Germany; 2 University of Leipzig, Department of Biology II, Johannisallee 21, 04103 Leipzig, Germany; 3 Georg-August University, Department of Forest Botany and Tree Physiology, Büsgenweg 2, 37077 Göttingen, Germany; 4 Max Planck Institute for Biogeochemistry, Hans-Knöll-Straße 10, 07745 Jena, Germany; 5 German Centre for Integrative Biodiversity Research (iDiv) Halle-Jena-Leipzig, Deutscher Platz 5e, 04103 Leipzig, Germany

## Abstract

Distance decay, the general reduction in similarity of community composition with increasing geographical distance, is known as predictor of spatial variation and distribution patterns of organisms. However, changes in fungal communities along environmental gradients are little known. Here we show that distance decays of soil-inhabiting and root-associated fungal assemblages differ, and identify explanatory environmental variables. High-throughput sequencing analysis of fungal communities of beech-dominated forests at three study sites across Germany shows that root-associated fungi are recruited from the soil fungal community. However, distance decay is substantially weaker in the root-associated than in the soil community. Variance partitioning of factors contributing to the observed distance decay patterns support the hypothesis that host trees stabilize the composition of root-associated fungi communities, relative to soil communities. Thus, they not only have selective impacts on associated communities, but also buffer effects of changes in microclimatic and environmental variables that directly influence fungal community composition.

A major goal of ecological research is to characterize processes responsible for spatial variation in organism communities^1^,^2^. Among other aspects there is increasing interest in elucidating drivers of soil fungal communities’ spatial turnover^3^,^4^,^5^. Knowledge of specific environmental factors’ effects on fungal communities is rapidly growing^6^,^7^,^8^,^9^. However, little is known about spatial variations in effects of such factors and ecological drivers on fungal communities, and potential modulations of their impacts in differing ecosystem compartments such as roots and the surrounding soil^9^,^10^,^11^.

The similarity of communities declines with increasing distance^12^. This “distance decay”^13^ could be driven by three main mechanisms. An intuitively obvious mechanism is that environmental conditions become increasingly different with increases in geographical distance, resulting in a niche-based community organization as species with different functional abilities are selectively recruited and form distinct communities^12^,^14^. The other two are modulation of dispersal rates of taxa by diverse barriers associated with landscape heterogeneity^15^, and dispersal limitations of organisms in homogenous landscapes^1^. Thus, similarity between communities can be affected by both stochastic dispersal and speciation processes, as recognized in the neutral theory^1^, and deterministic (e.g. niche- and dispersal-based) processes that vary among different organisms and ecosystems^12^,^13^.

Numerous environmental factors shape soil^16^,^17^,^18^ and root-associated^19^,^20^,^21^ fungal communities. However, it was recently reported that soil fungal community assemblies are strongly influenced by stochastic processes^22^. In temperate forests, the root-associated fungal communities (RAFC) mainly consist of ectomycorrhizal, saprotrophic, endophytic, or pathogenic fungi^9^,^23^. Members of these groups are host species dependent and some species are even host-specific^24^,^25^,^26^,^27^,^28^. Therefore, besides biotic and abiotic filters, which are common in community assemblies, RAFCs are also influenced by “host filters”^29^. However, fungi associated with roots are recruited from the surrounding soil fungal community (SFC)^30^, and these recruited components of the SFC depend on local environmental conditions, nutrient availability, plant nutrition and defense strategies, root structure and exudation^31^,^32^,^33^. Thus, the RAFC should presumably be less diverse and less taxa-rich than the surrounding SFC.

Despite evidence that the two fungal communities differ in habitat and diversity, it is unclear whether their spatial patterns are driven by similar or different processes, which limits our understanding of community shifts and their functional ecosystem-level effects. We have addressed this uncertainty by analyzing root-associated and soil fungal communities in 57 plots in three temperate forest areas dominated by European beech (*Fagus sylvatica* L.) across a north-south transect in Germany. We tested two hypotheses: that due to the strong plant control in its recruitment, RAFCs will be less affected by distance decay than SFCs, and hence environmental parameters (soil chemistry, soil texture, precipitation, temperature, vegetation and geographical location) will affect SFCs more than RAFCs.

## Results

### Root-associated fungi are mainly recruited from soil

In total, 931,320 sequences (237,610 from soil and 693,710 from roots) were obtained from 114 root and soil samples from 57 beech-dominated plots spread across three areas along a north-south transect across Germany. Subsequent quality filtering resulted in 454,641 sequences (172,926 from soil and 281,715 from roots). After removing plant sequences in the root dataset and chimeric sequences in both datasets, 249,746 sequences (169,820 from soil and 79,926 from roots) remained. The sequence read normalization procedure resulted in 95,760 sequences representing 1,415 reads per soil sample and 265 reads per root sample. Merger and further clustering of the two datasets resulted in 5,090 fungal operational taxonomic units (OTUs) including 3,528 rare OTUs (with ≤3 reads). Removal of the rare taxa had no significant effect on the composition of the fungal community (see materials and methods). Consequently, only the 1,562 abundant fungal OTUs were used to test our hypotheses.

**Figure 1:**
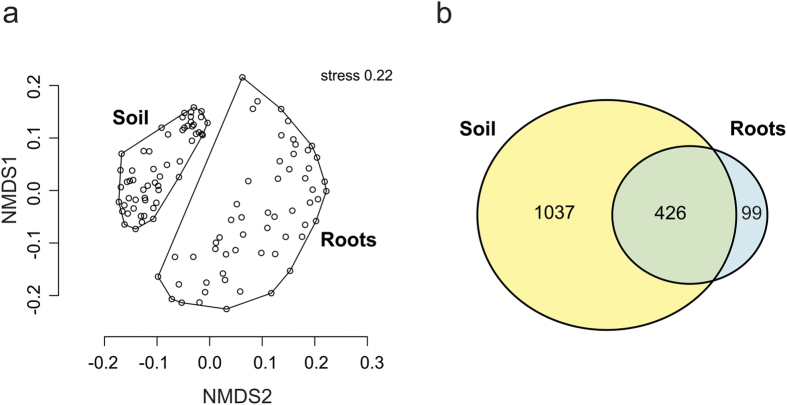
(**a**) Fungal community composition in the two compartments displayed using NMDS. Stress values represent percentages; (**b**) Venn diagram showing distributions of abundant fungal OTUs between the studied compartments (roots and soil) in all 57 beech-dominated plots.

Overall 88% of the 1,562 fungal OTUs obtained could be assigned to a fungal phylum. Members of the phylum Basidiomycota represented the majority of the fungal communities (Fig. S1). NMDS ordination analysis clearly separated root and soil fungal communities (Fig. 1a; ANOSIM; p < 0.001).

In the soil fungal community OTU richness and Shannon diversity were higher than in the root community (Student’s t-test; p < 0.001). Most of the root-associated fungal OTU were also found in the SFC (94%; 426 OTUs of the root community, corresponding to 27% of the soil community). In total, 1,037 OTUs (around 66%) were unique to soil and only 99 (6%) were unique to roots (Fig. 1b).

Approximately 50%, i.e. 781 OTUs could be assigned to the genus level. A total of 166 different fungal genera (Table S1) were identified. Prominent taxa like the ectomycorrhizal *Lactarius*, *Russula* and *Inocybe* and 60 additional fungal genera belonged to the shared fungal communities (Table S1). The root-associated fungal communities comprised only three unique genera identified as *Flagelloscypha*, *Paxillus* and *Cystolepiota* (Table S1). In the SFC 100 unique genera were found, in which *Oidiodendron*, *Cenococcum* and *Leotia* were the most abundant ones (Table S1).

### Distance decay functions of soil and root-associated fungal communities differ

**Figure 2:**
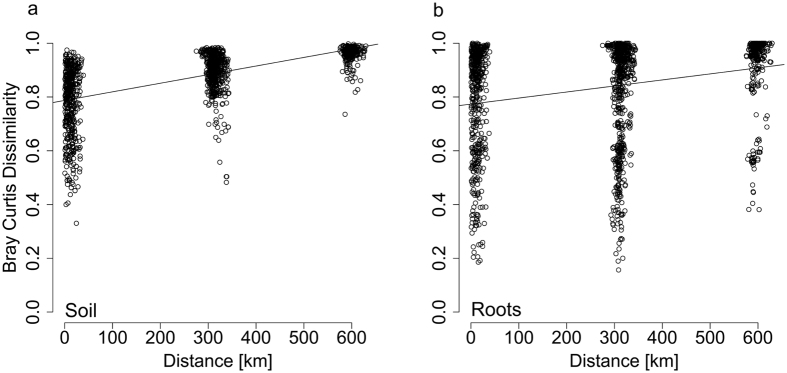
Correlation of Bray Curtis Dissimilarity with geographical distance between study plots for (**a**) soil and (**b**) root fungal communities.

Mantel correlation analysis of the impact of distance on the fungal communities derived from soil and roots using fungal community dissimilarity and geographic distance matrices showed increasing dissimilarities with increasing distance, i.e. distance decay (Fig. 2). Furthermore, slopes of the regression lines for the SFC (Fig. 2a) and RAFC (Fig. 2b) significantly differed (F = 15.93, degrees of freedom = 1, p < 0.001; p = 0.657 and 0.359, respectively). Accordingly, the SFC had greater composition dissimilarity among study plots than the RAFC (mean ± SE: 0.877 ± 0.003 and 0.837 ± 0.005, respectively; Wilcoxon test p = 0.001, Fig. S2). Thus, the SFC was clearly more affected by the distance decay than the RAFC.

### Impact of environmental parameters on the soil and root fungal communities

**Figure 3:**
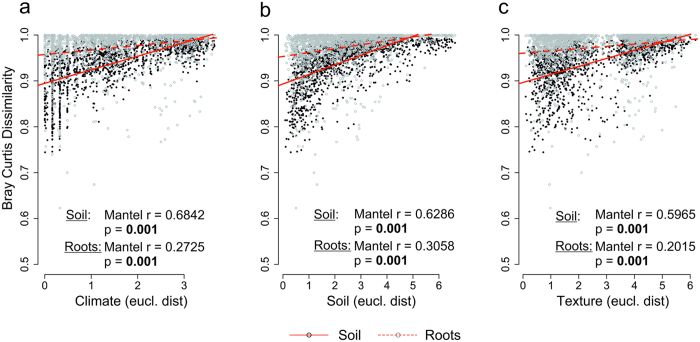
Bray Curtis Dissimilarity of fungal communities versus Euclidean distance of: (**a**) climate (mean annual temperature and annual precipitation); (**b**) soil chemical properties (organic and inorganic C, CN ratio and pH); and (**c**) soil texture (clay, fine and medium silt, fine and medium sand contents). Mantel r (−1 –1), indicator of effect direction: −1, negative effect; 0, no effect; 1, positive effect. p, significance value; values significant at p < 0.05 shown in bold.

Mantel correlation analysis was also applied after removing auto-correlated variables (Fig. S3) to examine effects of three groups of environmental variables, designated climate (mean annual temperature and annual precipitation), soil chemistry (organic and inorganic carbon contents, C/N ratio and pH) and soil texture (clay, fine and medium silt, fine and medium sand contents) on fungal communities. The composition of SFCs and RAFCs was significantly affected by changes in the abiotic environment (Fig. 3) as the dissimilarity of the fungal communities increased with increasing Euclidean distance in each of these three grouped variables. Distance-based redundancy analysis revealed significant effects of individual variables on both soil and root-associated fungal community composition (Table S2). Mantel r (Fig. 3) and F values (Table S2), respectively, were higher for soil fungal communities.

**Figure 4:**
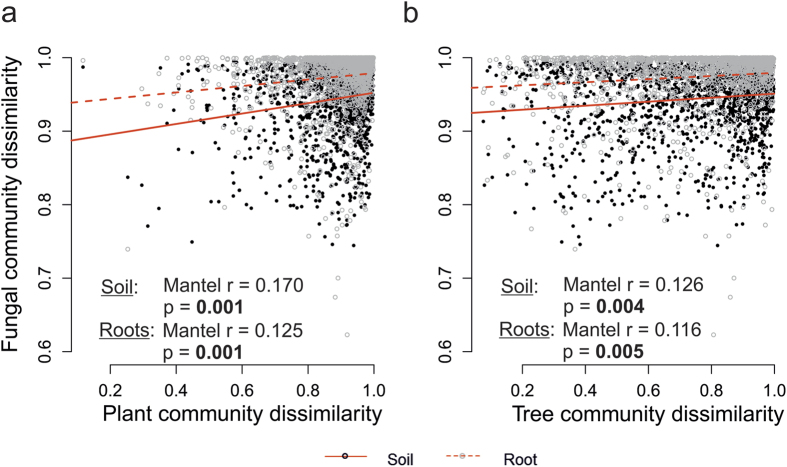
Bray Curtis dissimilarity of fungal communities versus Bray Curtis dissimilarity of (**a**) all vascular plants; (**b**) tree species; Mantel r (−1 –1), indicator of effect direction: −1, negative effect; 0, no effect; 1, positive effect. p, significance value; values significant at p < 0.05 shown in bold.

The total vegetation and tree species community affected fungal communities in soil and associated to roots significantly (Fig. 4). However, slopes indicated that SFC and RAFC are similarly shaped by vegetation (Fig. 4).

**Figure 5:**
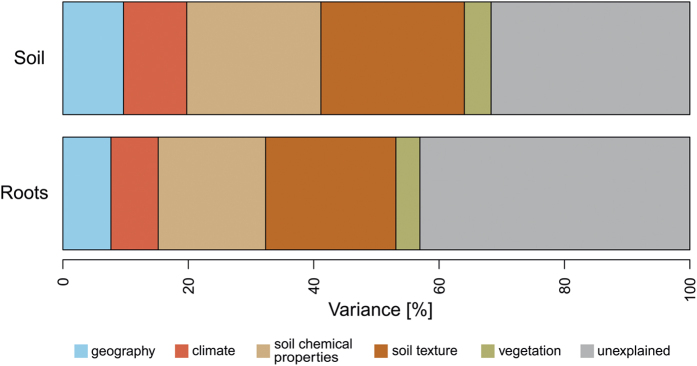
Variance partitioning of the fungal communities found in the soil and roots compartments, tested effector categories: geography, climate, soil chemical properties, soil texture and vegetation (defined in text).

Variance partitioning was applied to identify relative effects of the five categories of environmental variables (geographic location, climate, soil chemistry, soil texture and vegetation,) on the fungal communities (F values from ANOVA for SFC and RAFC: 1.5466 and 1.1961, respectively, p = 0.001 and degrees of freedom = 14 in both cases; Fig. 5). Among these parameters soil texture and soil chemistry explained the most variance of both soil and root-associated fungal communities, but 6% more of the SFC variance than the RAFC variance. Furthermore, the climatic variables and geographical position also explained more of the SFC variance than the RAFC variance, whereas for vegetation only 0.5% more variance is explained for the soil as compared to the root-associated fungal community. However, for root-associated fungal communities more variance remained unexplained compared to soil fungal communities (Fig. 5).

## Discussion

Distance decay, the general reduction in community similarity with increasing geographical distance^12^, is known to involve both intrinsic factors (such as the ability of taxa to disperse and niche selection) and neutral processes related to dispersal limitation and stochastic events^1^,^34^. Previous findings indicate that soil fungal community assembly may have a strong stochastic component^22^. However, our results support the hypothesis that RAFCs are less affected by distance decay than SFCs because they occupy particular habitats offered by host plants. Thus, we have identified a contextual dimension in such patterns, as relative strengths of influencing processes on terrestrial fungi appear to depend on their compartment (soil or roots). We detected distance decay patterns in both SFC and RAFC through simultaneous analysis of fungal sequences in the two compartments across a north-south gradient covering approximately 600 km in a temperate forest ecosystem in Germany, but observed effects were stronger in soil than in root associated communities (Fig. 2). Differences in distance decay among the two ecological compartments may be partly due to the lower species richness in the RAFC. Overall, in accordance with other studies^30^,^31^,^32^, we found that members of the RAFC were selectively recruited from the surrounding soil since almost all detected root-associated fungal OTUs were also found in the soil (Fig. 1b; Tables S1).

Furthermore, climate, soil chemical properties and soil texture all had weaker effects on RAFC than on SFC (Fig. 3), whereas the effect of vegetation was similar on fungal communities in both compartments (Fig. 4). This confirms our second hypothesis that habitats provided by individual host plants for RAFC have buffering effects. Differences in the steepness of regression slopes of SFC and RAFC dissimilarities against environmental variables show that the root habitat buffers effects of both chemical and physical changes in soil^35^. This could potentially increase SFC variability, through changing niches, while maintaining RAFC.

Environmental filtering reportedly influences fungal communities’ spatial patterns substantially^36^, but there are also indications that deterministic processes’ contributions may be weakened by hosts’ provision of habitats^10^,^37^. For example, neither distance decay nor strong effects of tested environmental factors have been detected in some examined communities of fungal endophytes^10^ and dead wood fungi^37^. However, as also previously reported^38^, variations of environmental conditions between the sampling areas contributed to the distance decay we observed, and variance partitioning revealed that area-specific environmental properties were more important than the geographical location. Generally, the measured parameters explained 68% of the SFC and 57% of the RAFC variance. The soil chemistry (pH, organic and inorganic carbon contents, and C/N ratio) accounted for ca. 21% and 17% of the explained variation in SFC and RAFC, respectively. Accordingly, previous studies have shown that soil pH can strongly affect soil fungal community composition in diverse ecosystems^9^,^17^,^39^, partly indirectly through effects on other soil properties, including availability of soil nutrients^40^. Similarly, the C/N ratio reportedly affects fungal communities in forest soils^7^,^17^,^39^. Soil texture accounted for 23% and 21% of the explained variance in the SFC and RAFC of the studied beech-dominated forest sites, respectively, in contrast to previous findings that soil silt and clay contents had no effect on fungal communities along a land-use gradient^16^. However, soil texture influences soil organic matter contents^41^, thereby affecting the fungal community structure^42^.

The climatic variables (annual precipitation and temperature) explained 10.1% and 7.5% of the SFC and RAFC variance, respectively, and the RAFC responded to changes in precipitation. These findings are consistent with expectations as precipitation and temperature are linked to soil moisture, which correlates with soil fungal community composition^11^,^43^. Furthermore, fungi have taxa-specific temperature optima in incubation experiments^44^,^45^, and under field conditions, temperature and soil moisture shape soils’ microbial communities^46^. Thus, together with geographic location (which explained 9.7% and 7.7% of the variance in SFC and RAFC, respectively), the environmental variables strongly contributed to the observed distance decay in both examined communities. Although it is known, that plant community composition affects associated communites^20^, the effect of vegetation on SFC (4.3%) and RAFC (3.8% explained variance) appeared to be comparable. Thus, unexplained variation (32% and 43% of the total variance for SFC and RAFC, respectively) could be attributed to numerous unmeasured variables, e.g. interactions with other below- and above-ground species^20^, amounts and profiles of root exudates^47^, or even anthropogenic effects^48^. However, the relative stability of interactions within the “beech root habitat” might be the main reason why the level of unexplained variance was more than 10% higher for RAFC than for SFC.

In conclusion, the significant difference in distance decay between the soil-inhabiting and root-associated fungal communities is consistent with dispersal limitation theory, and partly attributable to effects of spatial changes in soil properties. Consequently, no cosmopolitan distribution patterns were detected in either root-associated or soil fungal communities. Since our study was limited to three biogeographic areas, further investigations are required to address the assembly rules of fungal communities across larger scales including temporal patterns. Moreover, our results also indicate the importance of the host tree species and future studies need to address the interactive effects of soil physico-chemical properties, host plant root traits and exudation patterns, to improve mechanistic and functional understanding of the rhizosphere microbial communication and shifts in community composition. In general our results provide a stimulating insight for new ideas of theoretical models related to fungal meta-communities, biogeography and landscape ecology.

## Material and Methods

### Study areas

The study was performed in the Biodiversity Exploratories: three study areas along a 600 km geographical transect, located in the south-west (Swabian Alb), centre (Hainich-Dün) and north-east (Schorfheide-Chorin) of Germany^49^. Beside topo-geographical variations they also differ in geology and climate (for details see Table S3^49^,^50^). Since ectomycorrhizal fungal communities are particularly known for host tree preferences^17^,^26^ we chose 19 widely spread, beech-dominated, 100 × 100 m plots per study site to exclude effects of fungal host preferences in our analyses (Fig. S4, Table S4).

### Soil and root sampling

In early May 2011 soil was sampled from each of the 57 experimental plots at all study areas, by collecting 14 soil cores of 5 cm diameter and 10 cm depth (after removing organic litter) at points 1, 7, 13, 19, 31 and 37 m from starting points of two 40 m transects (north-south and east-west). The cores obtained from each plot were sieved (mesh size, 2 mm), mixed into a composite sample, and 50 g of each composite sample was stored at −80 °C for molecular analysis. After sieving, the root samples were collected and pooled separately. About 2 g samples of roots were washed in water that had been deionised and sterilised using a USF Seralpur System (Seral, Ransbach-Baumbach, Germany) with a DCF CHS92DE Delta Supor Filter (Pall Cooperations, Washington, NY, USA) at 4 °C, then frozen in liquid nitrogen. The root samples were stored temporarily at −80 °C then freeze-dried using a P4K-S System (Dieter Piatkowski Forschungsgeräte, Munich, Germany) and PK4D vacuum pump (ILMVAC GmbH, Ilmenau, Germany) starting at −60 °C rising to −20 °C for four days and finally stored at room temperature.

### DNA extraction, amplicon library preparation and pyrosequencing

#### Soil

Microbial genomic DNA was extracted from two independent 0.5 g frozen subsamples of each composite soil sample using MoBio Power Soil DNA isolation kit (MoBio Laboratories, Carlsbad, CA, USA) following the manufacturer’s recommendations. The two soil DNA extracts per sample were pooled and their DNA concentrations were quantified using a NanoDrop UV-Vis spectrophotometer (Peqlab Biotechnologie GmbH, Erlangen, Germany). Although the ITS primers are known for their taxonomic bias towards Ascomycota and Basidiomycota by favoring certain sequence lengths during PCR, they are the established barcodes for identifying fungal communities from environmental samples^51^. Furthermore there is a dominance of ITS sequences in public fungal databases^52^. Hence, the fungal ITS rDNA barcode region was amplified using custom ITS1F primers^53^ containing Roche 454 pyrosequencing adaptor A and the universal primer ITS4^54^ containing Roche 454 pyrosequencing adaptor B and a sample-specific MID. The PCR reaction mixtures (50 μl) contained 1 μl DNA template (7–15 ng), 25 μl Go Taq Green Master mix (Promega, Mannheim, Germany) and 1 μl of each of the ITS region-specific primers (25 pmol). Touchdown PCR was performed under the following conditions: initial denaturation for 5 min at 95 °C followed by: (1) 10 cycles of 94 °C for 30 sec, 60–50 °C for 45 sec (−1 °C per cycle) and 72 °C for 2 min; and (2) 30 cycles of 94 °C for 30 sec, 50 °C for 45 sec and 72 °C for 2 min with a final extension step of 10 min^7^. All samples were amplified in triplicate, purified using Qiagen gel extraction kit (Qiagen, Hilden, Germany), their DNA concentrations were measured using Cary Eclipse fluorescence spectrophotometer (Agilent Technologies, Waldbronn, Germany) and pooled equimolarly. The amplicons were unidirectionally pyrosequenced from the ITS4 end using a 454 titanium amplicon sequencing kit and the GS-FLX + 454 pyrosequencer (Roche, Mannheim, Germany) at the Department of Soil Ecology, Helmholtz Centre of Environmental Research (UFZ, Halle, Germany).

#### Roots

The freeze-dried root samples were ground in a MM2 ball mill (Retsch, Haan, Germany), and DNA was extracted from them following the same procedure as for soil samples using the MoBio Power Soil DNA isolation kit. The fungal ITS rDNA fragment was amplified using the ITS3 primer pair^54^ containing Roche 454 pyrosequencing adaptor A and the universal primer ITS4 54 containing Roche 454 pyrosequencing adaptor B and a sample-specific MID. The PCR mixtures (50 μl) contained 35 μl sterile nuclease-free water, 5 μl 10xPfu PCR buffer with MgSO_4_, 1 μl dNTP Mix (10 mM) 0.5 μl Pfu DNA polymerase (2.5 u/μl; all reagents from Thermo Fisher Scientific, Waltham, MA, USA), 2 μl of each primer (10 μl) and 4 μl of DNA template (diluted 1:10). We performed touchdown PCR following the same procedures as for soil DNA. All samples were amplified in triplicate, and products were purified using the Qiagen gel extraction, as above, following the manufacturer’s instructions. After the amplification DNA concentrations were measured using a NanoDrop UV-Vis spectrophotometer and the PCR products of each triplicate were pooled equimolarly. A unidirectional pyrosequencing from the ITS4 end of the amplicons was performed using a 454 titanium amplicon sequencing kit and the Roche GS-FLX 454 pyrosequencer (Roche, Mannheim, Germany) following the instructions of the manufacturer at the Göttingen Genomics Laboratory (Germany).

### Bioinformatic analyses and data normalization

The soil- and root-based 454 ITS sequences were processed and quality-filtered on multiple levels using MOTHUR software^55^ as previously described^17^. Briefly, in the initial filtering step, sequences with ambiguous bases, homo-polymers and primer differences of more than eight bases were removed. Simultaneously all primer and barcode sequences were discarded. At the same time, sequence reads with a quality score lower than 20 and read length less than 300 bp were removed, using the keepfirst 300 bp command and thereby chopping at least 50 bp of the sequence end to remove sequencing noise. This resulted in a 300 bp sequence read fragment covering the ITS2 region in both datasets. We detected strong presence of plant ITS sequences in the root dataset, so we applied a virtual ecoPCR^51^,^56^ with the primers ITS1F and ITS3^54^ (allowing two mismatches) with lengths between 100 and 800 nt. First, all genome sequence scan, high-throughput genome sequencing and standard sequence classes from plants were retrieved from the EMBL^57^ release 118 of December 2013. Then, whole genome sequences of plants were retrieved from the same EMBL release, and one entry per species name was conserved for further analyses. The resulting custom database with amplified plant sequences was used to filter out plant sequences from our root dataset. After a chimera check of both datasets using the uchime algorithm^58^ implemented in MOTHUR^55^, potential chimeric sequences were removed. The range of numbers of sequence reads differed substantially between the root and soil datasets (265–9314 and 3265–3141 per sample, respectively).

A crucial requirement in any comparison of microbial community datasets is sampling to species saturation or, if this is not possible, using the same or normalized sample numbers (in this context, sequence reads). We decided to normalize the datasets based on rarefaction curves rather than sample numbers because we expected the RAFC to be a subset of the SFC^30^, and thus less species-rich. The smallest sample in the root dataset had 265 sequence reads (from Hainich-Dün, HEW06). Thus, we calculated numbers of fungal OTUs at 265 sequence reads and estimated OTU numbers at saturation with the Chao1 index using R (version 3.1.1)^59^ and the ‘estimateR’ function of the vegan package (version 2.0–10)^60^. Based on the rule of three, we then obtained the percentage of the index for 265 sequences (≈35%; see Table S5). The Chao1 estimation was repeated for the smallest soil sample, obtained from Schorfheide-Chorin (SEW47), in the same manner to obtain the number of sequences at 35% of the asymptotic value (Fig. S5, Table S5). With the ‘rarefaction’ function provided by Jenna Jacobs (http://www.jennajacobs.org/R/rarefaction.html) we obtained a table with values for each curve, so we could retrieve associated sequence values (1415 sequences per sample SEW47_soil) for our calculated numbers of OTU (169.63 SEW47_soil). The following formulas summarize our procedure:


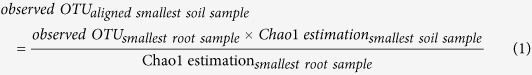


In our specific case:


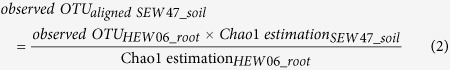


Finally, the normalization process was repeated 10 times for both soil (1415 sequences per sample) and roots (265 sequences per sample) by random removal of sequence reads using the subsample command as implemented in MOTHUR^55^. Merging processes led to 10 datasets combining fungal sequences from soil and roots. The sequences from the respective datasets were clustered into OTUs using cd-hit-est^61^ at a threshold of 97% pairwise identity. Taxonomic assignment of the representative sequences of the OTUs was done by the classify.seq command of MOTHUR^55^ using the UNITE fungal ITS reference database (version 6)^52^.

In order to select the most appropriate dataset for our analysis, we performed Procrustes-based tests of the ten datasets, by applying the protest function^62^ of the vegan package^60^ in R^59^ for pairwise comparisons of the correlation between the NMDS ordinations derived from a log-transformed abundance matrix. For each replicate the sums of squares were summed from the nine comparisons and the one with the lowest difference from the other replicates (lowest sum of squares) was selected as a representative dataset and used for further statistical analysis (Procrustes correlation coefficients, sums of squares and p-values are presented in Table S6).

### Environmental parameters

#### Soil chemical properties and soil texture

All soil analyses were performed with air-dried samples sieved at <2 mm. The pH of supernatants of duplicate suspensions of the soil samples in 0.01 M CaCl_2_ (1:2.5) was determined using a glass electrode. Ground mineral soil samples (<100 μm) were analyzed for total carbon (C) and nitrogen (N) by dry combustion with a Vario Max CN analyzer (Elementar Analysensysteme GmbH, Hanau, Germany). After removing organic C by combusting samples for 16 h at 450 °C, inorganic C was determined using the same method. Organic C concentrations were then calculated from the differences between total and inorganic C concentrations^50^.

Soil texture was determined using the pipette method^63^ after removing organic matter.

#### Climate data

Air temperature and precipitation data were extracted, for grid cells for each of our study plots, from the Bioclim subset of the Wordclim dataset^64^. These are both gridded datasets with a spatial resolution of 30 arc seconds obtained by interpolating averaged values recorded at climate stations between 1950 and 2000.

#### Vegetation

Forest plant community inventory data (including all vascular plants) was extracted for each of our study plots^65^. Stand density values derived from the Silvicultural Management Index (SMI)^66^ for all study plots.

### Statistics

Statistical analyses were performed using R software (version 3.1.1)^59^. To define the data matrix for our statistical analyses, we first tested the effect of removing rare fungal taxa on community composition. To assess the influence of rare fungal OTUs (represented by ≤3 reads), we calculated the non-metric multidimensional scaling (NMDS) ordination with 20 random starts from the dataset both with all OTUs and with only the abundant fungal OTUs (OTUs represented by >3 reads). The congruence between the two ordination sets was tested by Procrustes correlation analysis using the protest function^62^ of the R package vegan^60^ with 999 permutations. We found that fungal community composition was not significantly affected by the presence or absence of rare fungal OTU (Procrustes correlation coefficient = 0.9964; *p* < 0.001, suggesting nearly identical ordination). We also tested the need for re-normalization of the abundant fungal OTU data matrix. We compared the congruence of the NMDS plots based on the dominant fungal OTU data matrix and on a re-normalized abundant OTU data matrix using Procrustes correlation analysis. We found that this normalization step did not affect the fungal community composition (Procrustes correlation coefficient = 0.9772; *p* < 0.001). Hence, all subsequent analyses were performed using the fungal community matrix excluding singletons, doubletons and tripletons.

The proportions of unique OTUs and those shared by the soil and root compartments were visualized as Venn diagrams using the R package VennDiagram^67^. Fungal OTU diversity was assessed by calculating Shannon-Wiener diversity indices^68^ using the diversity function in vegan^60^. Differences in fungal diversity and OTU richness were compared using Student’s t-Test assuming unequal variance (F-Test, p < 0.05) and incorporating Jarque-Bera test for normality under usage of the R package fBasics^69^. Relationships of fungal communities of soil and roots were visualized using NMDS on the basis of a Bray-Curtis distance matrix and 30 random starts using the metaMDS and ordihull functions of the vegan package^60^.

To examine spatial variation of the fungal community and relationships of its composition to environmental factors, we computed Mantel statistics^70^ using the vegan package^60^. This approach tests associations between distance matrices. Dissimilarity matrices were calculated for the fungal OTU matrices and the vegetation (all vascular plants and just tree species) using Bray-Curtis distances. Regression slopes of soil and fungal communities with distance were compared by the function “Comparison of regression lines” in Statgraphics Centurion XVI (Statpoint Technologies, Warrenton, VI, USA). To test for co-linearity of the environmental variables, we applied Spearman-rank correlation tests using the R package Hmisc^71^. Environmental variables with a Spearman rank correlation coefficient p > 0.8 were removed and excluded from further analysis. The effects of the remaining z-transformed environmental parameters on the fungal community Bray-Curtis dissimilarities were tested using distance based redundancy analysis (dbRDA) as implemented in the R package vegan^60^. Distance matrices were constructed using the Euclidean distances from the remaining and z-transformed environmental parameters and sorted into three effect groups. These were: climate (annual mean temperature and annual precipitation); soil chemical properties (organic and inorganic carbon contents, C to N ratio and pH); and soil texture (clay, fine and medium silt, fine and medium sand contents). A geographical distance matrix consisting of untransformed distances between the observed plots (in km) was constructed, then Mantel statistics were calculated for all pairs of distance matrices against the OTU dissimilarity matrices using the default setting of 999 permutations.

In order to understand the relative contribution of geographical location, vegetation, climatic and soil conditions in shaping the fungal communities in soil and roots we performed variance partitioning. For this purpose we used the capscale command as implemented in vegan^60^. Values of all environmental factors, including latitude and longitude of each plot, were z-transformed.

## Additional Information

**Accession code:** The raw ITS rDNA sequences have been deposited in the National Center for Biotechnology Information (NCBI) Sequence Read Archive (SRA) under study accession number SRP070568 (PRJNA312066).

**How to cite this article**: Goldmann, K. *et al*. Divergent habitat filtering of root and soil fungal communities in temperate beech forests. *Sci. Rep.* **6**, 31439; doi: 10.1038/srep31439 (2016).

## Supplementary Material

Supplementary Information
