# Supplementary material for: Divergent habitat filtering of root and soil fungal communities in temperate beech forests
Source: Sci Rep. 2016 Aug 11;6:31439. doi: 10.1038/srep31439 (PMC4980589; doi:10.1038/srep31439)
Supplement: Supplementary Information [file srep31439-s1.doc]

# **Divergent habitat filtering of root and soil fungal communities in temperate beech forests**

Kezia Goldmann, Kristina Schröter, Rodica Pena, Ingo Schöning, Marion Schrumpf, François Buscot, Andrea Polle and Tesfaye Wubet

**Supplementary**

**Supplemental figures**


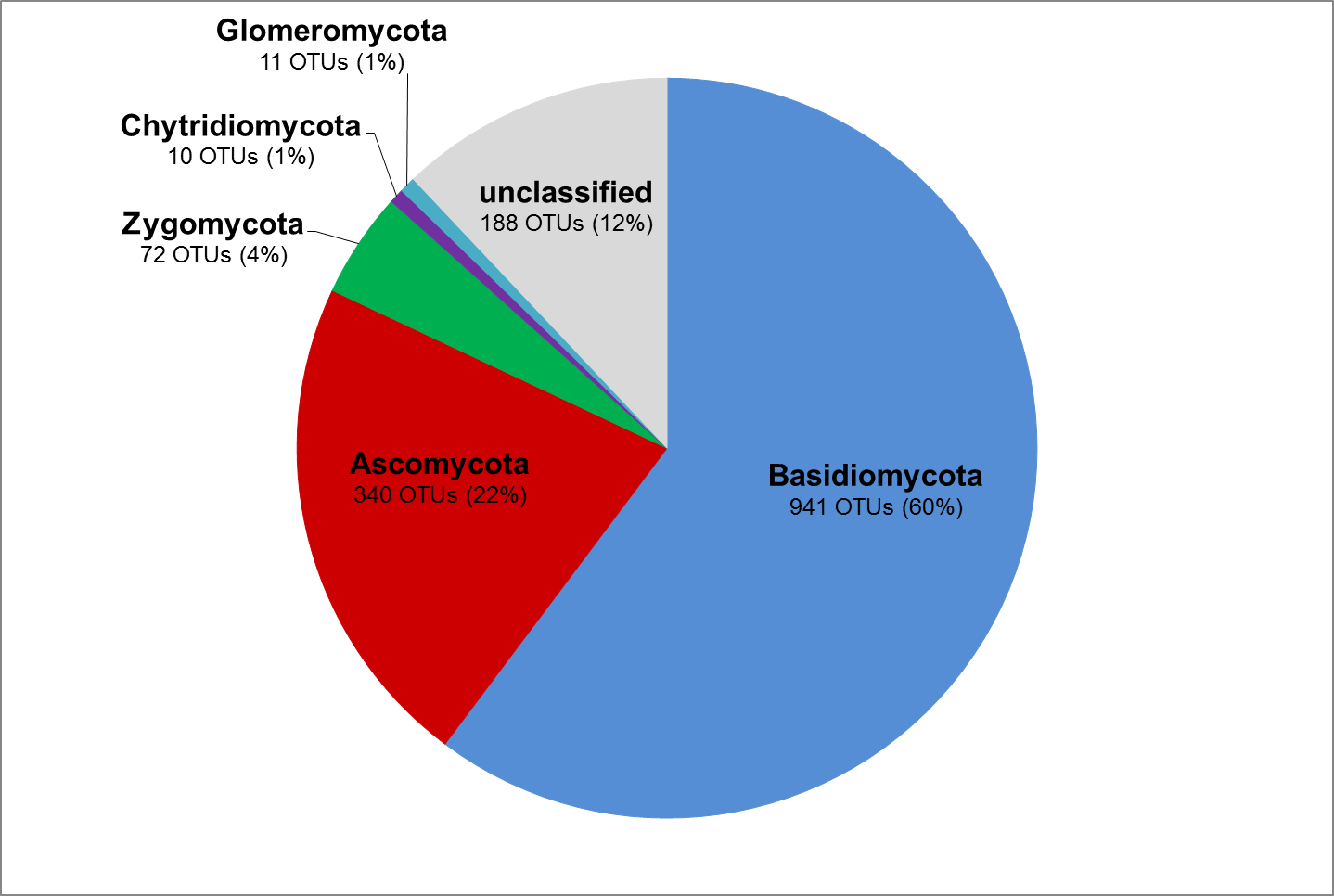


**Figure S1|** Taxonomic assignment at fungal phylum level. Numbers display the amount of fungal OTU assigned, in brackets are corresponding percentages.

.
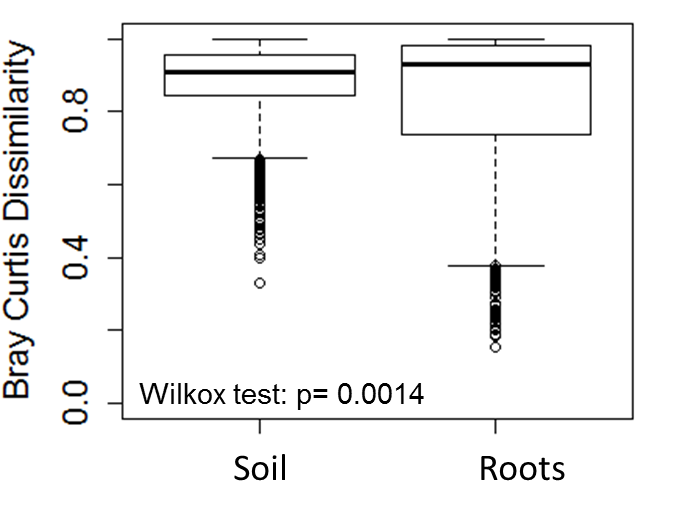


**Figure S2|** Boxplot of Bray Curtis dissimilarities of fungal communities between beech-dominated plots in the soil and root compartments.


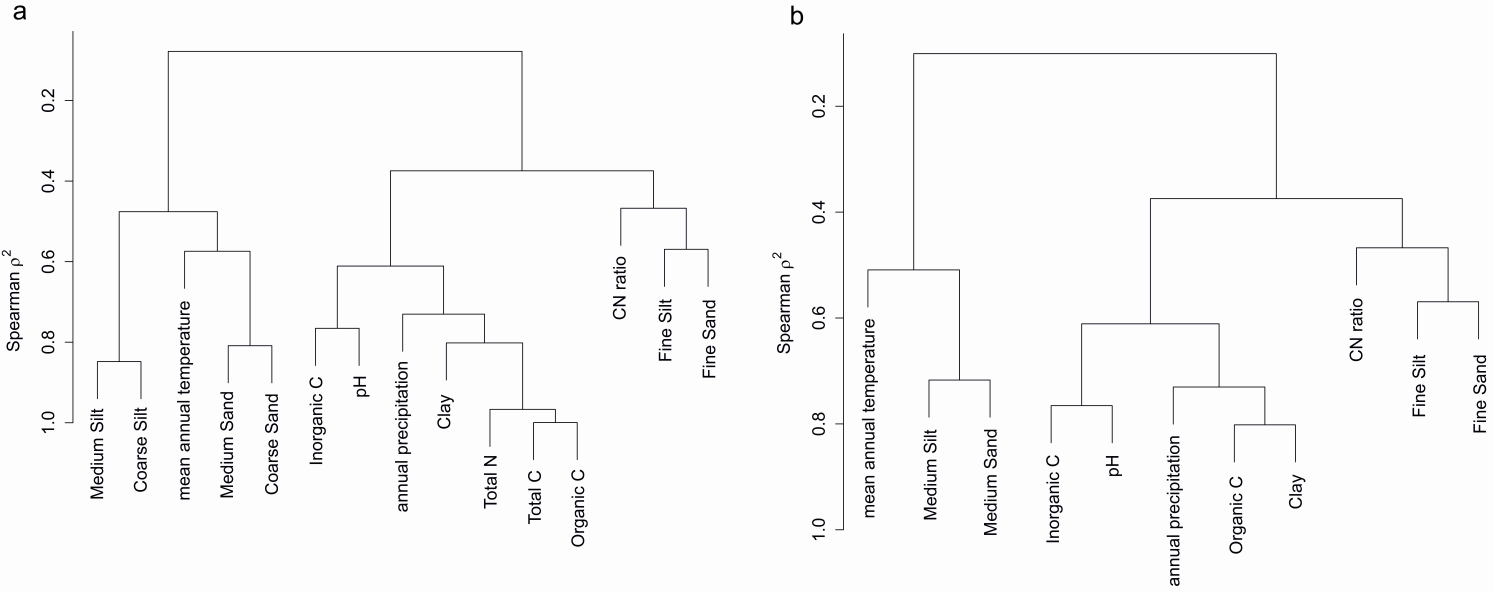


**Figure S3|** Spearman rank correlation tests of co-linearity of environmental variables: a) full model including all variables; b) reduced model, including variables with Spearman ρ² ≤ 0.8.


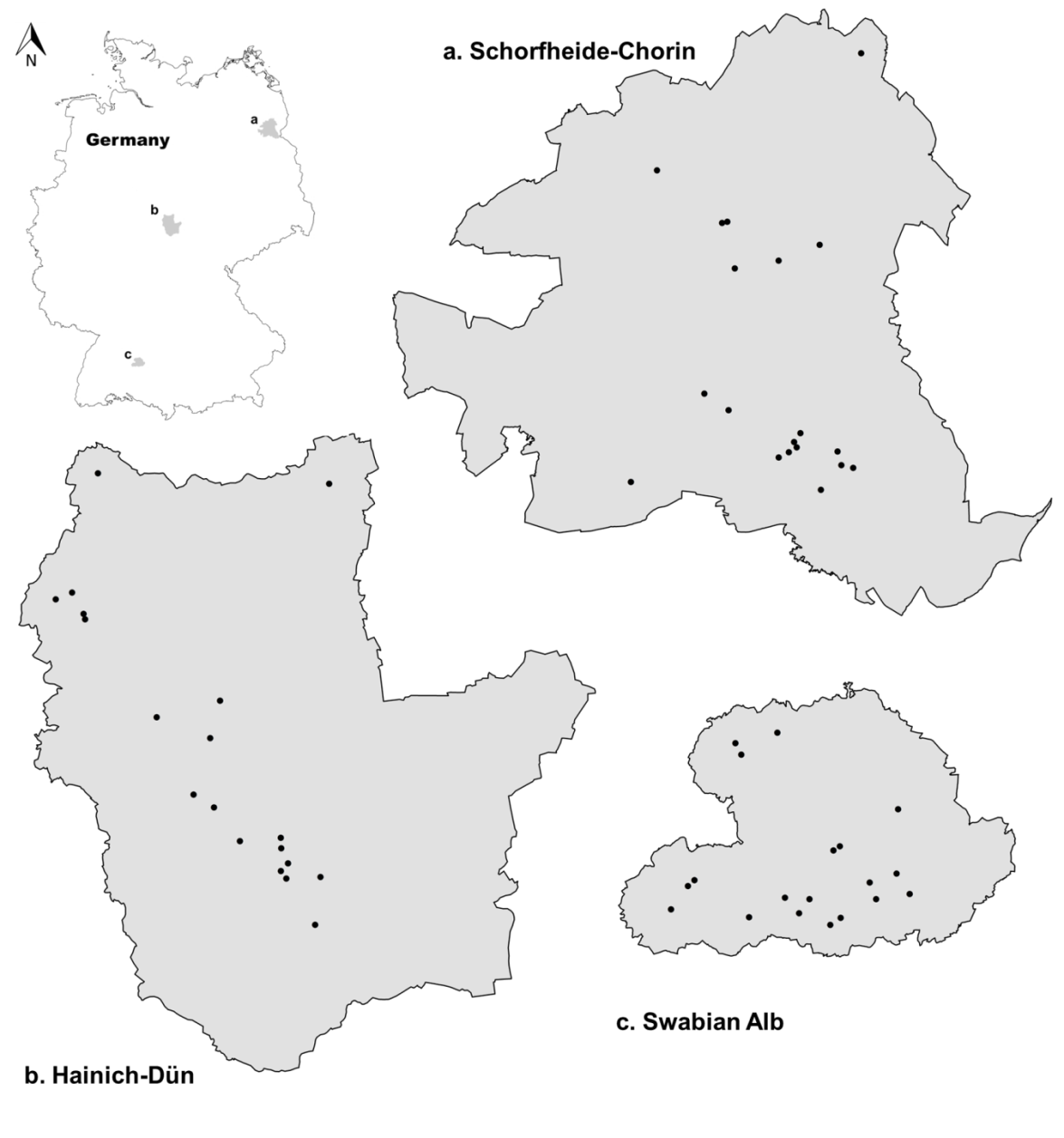


Figure S4| Overview of studied beech forest stands across the three areas of the German Biodiversity Exploratories. Each dot represents a 100 m x 100 m forest area. The scale for single sites is 1:250,000. Figure generated using ArcCatalog (ESRI (Environmental Systems Research Institute) 2015, ArcGIS Desktop: Version 10.3.1., Redlands, CA, USA).


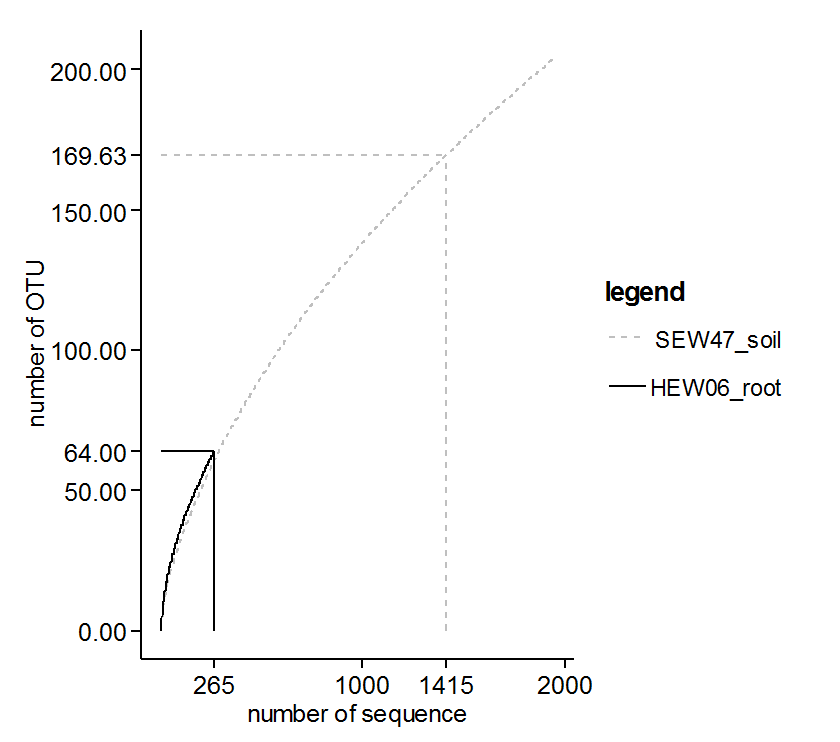


Figure S5| Rarefaction curves based on the Chao1 saturation estimator for roots and equivalent curve for setting the threshold for normalization.

**Supplemental tables**

**Table S1|** Overview of 166 different fungal genera, relative abundance values and compartment of appearance (shared community, SFC or RAFC).

| **Fungal genus** | **relative abundance** | **compartment** |
| --- | --- | --- |
| *Lactarius* | 25.6353 | Shared |
| *Russula* | 16.8558 | Shared |
| *Inocybe* | 5.0738 | Shared |
| *Mortierella* | 3.4605 | Shared |
| *Hygrophorus* | 2.5849 | Shared |
| *Boletus* | 2.1189 | Shared |
| *Piloderma* | 2.0899 | Shared |
| *Genea* | 1.9698 | Shared |
| *Cryptococcus* | 1.9037 | Shared |
| *Tomentella* | 1.5475 | Shared |
| *Elaphomyces* | 1.4323 | Shared |
| *Cortinarius* | 1.2784 | Shared |
| *Lactifluus* | 1.1894 | Shared |
| *Mycena* | 1.0762 | Shared |
| *Sebacina* | 0.9349 | Shared |
| *Amanita* | 0.8719 | Shared |
| *Tuber* | 0.8247 | Shared |
| *Tricholoma* | 0.8204 | Shared |
| *Armillaria* | 0.7873 | Shared |
| *Clavulina* | 0.7024 | Shared |
| *Thelephora* | 0.5520 | Shared |
| *Tarzetta* | 0.4067 | Shared |
| *Melanogaster* | 0.3337 | Shared |
| *Geminibasidium* | 0.2611 | Shared |
| *Agrocybe* | 0.2595 | Shared |
| *Entoloma* | 0.2442 | Shared |
| *Tylospora* | 0.2312 | Shared |
| *Hymenogaster* | 0.2010 | Shared |
| *Protoglossum* | 0.1755 | Shared |
| *Scopuloides* | 0.1703 | Shared |
| *Gymnomyces* | 0.1312 | Shared |
| *Pseudotomentella* | 0.1280 | Shared |
| *Xenasmatella* | 0.1197 | Shared |
| *Humaria* | 0.1147 | Shared |
| *Octaviania* | 0.1064 | Shared |
| *Amphinema* | 0.0975 | Shared |
| *Membranomyces* | 0.0848 | Shared |
| *Ramariopsis* | 0.0826 | Shared |
| *Lycoperdon* | 0.0814 | Shared |
| *Umbelopsis* | 0.0695 | Shared |
| *Malassezia* | 0.0564 | Shared |
| *Laccaria* | 0.0544 | Shared |
| *Otidea* | 0.0420 | Shared |
| *Rhizophydium* | 0.0407 | Shared |
| *Paecilomyces* | 0.0388 | Shared |
| *Physisporinus* | 0.0341 | Shared |
| *Leucosporidiella* | 0.0263 | Shared |
| *Acremonium* | 0.0214 | Shared |
| *Gymnopus* | 0.0204 | Shared |
| *Delicatula* | 0.0203 | Shared |
| *Terfezia* | 0.0195 | Shared |
| *Hydnotrya* | 0.0192 | Shared |
| *Macowanites* | 0.0189 | Shared |
| *Cephalotrichum* | 0.0179 | Shared |
| *Gliomastix* | 0.0152 | Shared |
| *Naucoria* | 0.0137 | Shared |
| *Clitocybe* | 0.0128 | Shared |
| *Megacollybia* | 0.0120 | Shared |
| *Ramichloridium* | 0.0119 | Shared |
| *Verticillium* | 0.0107 | Shared |
| *Tomentellopsis* | 0.0061 | Shared |
| *Anthracobia* | 0.0061 | Shared |
| *Amauroascus* | 0.0060 | Shared |
| *Oidiodendron* | 0.1774 | Soil |
| *Cenococcum* | 0.1495 | Soil |
| *Leotia* | 0.1335 | Soil |
| *Penicillium* | 0.1115 | Soil |
| *Microglossum* | 0.0963 | Soil |
| *Trichoderma* | 0.0836 | Soil |
| *Hypomyces* | 0.0802 | Soil |
| *Sistotrema* | 0.0726 | Soil |
| *Trechispora* | 0.0659 | Soil |
| *Leptodontidium* | 0.0620 | Soil |
| *Archaeospora* | 0.0591 | Soil |
| *Hygrocybe* | 0.0558 | Soil |
| *Dictyochaeta* | 0.0543 | Soil |
| *Exophiala* | 0.0523 | Soil |
| *Nectria* | 0.0518 | Soil |
| *Tephrocybe* | 0.0436 | Soil |
| *Meliniomyces* | 0.0340 | Soil |
| *Syzygospora* | 0.0275 | Soil |
| *Helvella* | 0.0274 | Soil |
| *Pochonia* | 0.0268 | Soil |
| *Scleroderma* | 0.0267 | Soil |
| *Clavaria* | 0.0237 | Soil |
| *Coprinellus* | 0.0209 | Soil |
| *Zalerion* | 0.0206 | Soil |
| *Craterellus* | 0.0199 | Soil |
| *Tetracladium* | 0.0187 | Soil |
| *Volutella* | 0.0187 | Soil |
| *Pseudogymnoascus* | 0.0177 | Soil |
| *Rhodotorula* | 0.0158 | Soil |
| *Preussia* | 0.0150 | Soil |
| *Pseudeurotium* | 0.0148 | Soil |
| *Gyroporus* | 0.0147 | Soil |
| *Echinoderma* | 0.0141 | Soil |
| *Archaeorhizomyces* | 0.0141 | Soil |
| *Scutellinia* | 0.0133 | Soil |
| *Resinicium* | 0.0127 | Soil |
| *Melanoleuca* | 0.0127 | Soil |
| *Botryobasidium* | 0.0127 | Soil |
| *Infundibulicybe* | 0.0123 | Soil |
| *Hypholoma* | 0.0120 | Soil |
| *Minutisphaera* | 0.0119 | Soil |
| *Itersonilia* | 0.0113 | Soil |
| *Trichosporon* | 0.0111 | Soil |
| *Scytalidium* | 0.0111 | Soil |
| *Phialocephala* | 0.0105 | Soil |
| *Conocybe* | 0.0098 | Soil |
| *Hypocrea* | 0.0090 | Soil |
| *Pseudocraterellus* | 0.0089 | Soil |
| *Marasmiellus* | 0.0088 | Soil |
| *Camarophyllopsis* | 0.0082 | Soil |
| *Rhodocybe* | 0.0081 | Soil |
| *Schizoblastosporion* | 0.0081 | Soil |
| *Cladosporium* | 0.0075 | Soil |
| *Xylaria* | 0.0075 | Soil |
| *Wardomyces* | 0.0074 | Soil |
| *Hymenopellis* | 0.0074 | Soil |
| *Adisciso* | 0.0074 | Soil |
| *Powellomyces* | 0.0060 | Soil |
| *Leohumicola* | 0.0060 | Soil |
| *Kretzschmaria* | 0.0058 | Soil |
| *Thelebolus* | 0.0054 | Soil |
| *Lasiosphaeris* | 0.0053 | Soil |
| *Chalara* | 0.0053 | Soil |
| *Chloridium* | 0.0053 | Soil |
| *Clavariadelphus* | 0.0053 | Soil |
| *Pluteus* | 0.0053 | Soil |
| *Phialophora* | 0.0052 | Soil |
| *Metarhizium* | 0.0052 | Soil |
| *Menispora* | 0.0052 | Soil |
| *Sporormia* | 0.0046 | Soil |
| *Ramsbottomia* | 0.0046 | Soil |
| *Heterobasidion* | 0.0045 | Soil |
| *Chaetosphaeria* | 0.0045 | Soil |
| *Hyphoderma* | 0.0045 | Soil |
| *Podospora* | 0.0044 | Soil |
| *Phylloporus* | 0.0044 | Soil |
| *Aspergillus* | 0.0044 | Soil |
| *Gyoerffyella* | 0.0044 | Soil |
| *Parasola* | 0.0038 | Soil |
| *Mastigobasidium* | 0.0038 | Soil |
| *Coprinus* | 0.0038 | Soil |
| *Hyalodendriella* | 0.0037 | Soil |
| *Rhizophlyctis* | 0.0037 | Soil |
| *Zignoella* | 0.0037 | Soil |
| *Lepiota* | 0.0037 | Soil |
| *Dactylellina* | 0.0037 | Soil |
| *Phallus* | 0.0037 | Soil |
| *Chlorophyllum* | 0.0031 | Soil |
| *Ilyonectria* | 0.0031 | Soil |
| *Hebeloma* | 0.0030 | Soil |
| *Lecythophora* | 0.0030 | Soil |
| *Bionectria* | 0.0030 | Soil |
| *Psathyrella* | 0.0030 | Soil |
| *Pyrenochaeta* | 0.0030 | Soil |
| *Cadophora* | 0.0030 | Soil |
| *Mycosphaerella* | 0.0030 | Soil |
| *Parmelia* | 0.0030 | Soil |
| *Lachnum* | 0.0030 | Soil |
| *Asterotremella* | 0.0029 | Soil |
| *Sporothrix* | 0.0029 | Soil |
| *Cystolepiota* | 0.0352 | Roots |
| *Paxillus* | 0.0244 | Roots |
| *Flagelloscypha* | 0.0165 | Roots |

Table S2| Results of distance-based redundancy analysis of the Bray Curtis fungal dissimilarity matrix versus environmental factors. Df – degrees of freedom, F statistics–mean of the within group variances, p, significance value; values significant at p < 0.05 shown in bold.

|  |  | Soil fungal community | | Root-associated fungal community | |
| --- | --- | --- | --- | --- | --- |
|  | Df | F | p | F | p |
| **Annual mean temperature** | 1 | 4.9983 | **0.001** | 1.9498 | **0.001** |
| **Annual precipitation** | 1 | 4.7947 | **0.001** | 2.3742 | **0.001** |
| **Inorganic carbon** | 1 | 3.0031 | **0.001** | 1.7028 | **0.001** |
| **Organic carbon** | 1 | 3.8944 | **0.001** | 2.1548 | **0.001** |
| **CN ratio** | 1 | 5.17 | **0.001** | 2.0864 | **0.001** |
| **pH** | 1 | 4.8053 | **0.001** | 2.3768 | **0.001** |
| **Clay** | 1 | 4.9374 | **0.001** | 2.3246 | **0.001** |
| **Fine silt** | 1 | 4.6936 | **0.001** | 2.0487 | **0.001** |
| **Medium silt** | 1 | 4.3336 | **0.001** | 1.7787 | **0.001** |
| **Fine sand** | 1 | 4.2709 | **0.001** | 2.1106 | **0.001** |
| **Medium sand** | 1 | 5.1032 | **0.001** | 2.0038 | **0.001** |
| **Forest stand density^*^** | 1 | 1.0523 | 0.242 | 0.9732 | 0.584 |

^*^SMId^1^

**Table S3|** Main geographical and environmental characters of the three Biodiversity Exploratories^2,3^(modified).

|  | **Schwäbische Alb** | **Hainich-Dün** | **Schorfheide-Chorin** |
| --- | --- | --- | --- |
| **Abbreviation** | ALB | HAI | SCH |
| **Location** | South West Germany | Central Germany | North East Germany |
| **Coordinates precise** | lat= 48.3639617-48.5000527;  lon= 9.22239205-9.50193186 | lat= 51.0460522-51.369932 ;  lon= 10.207728-10.5340491 | lat= 52.8619726-53.1922476;  lon= 13.6329537-14.0017904 |
| **Size** [km²] | ∼422 | ∼1300 | ∼1300 |
| **Geology** | Calcareous bedrock with karst phenomena | Calcareous bedrock | Young glacial landscape |
| **Soil type in forests** | Cambisol (eutric)-Leptosol | Luvisol | Cambisol (dystric) |
| **Annual mean temperature** [°C] | 6–7 | 6.5–8 | 8–8.5 |
| **Annual mean precipitation** [mm] | 700–1000 | 500–800 | 500–600 |
| **Altitude a.s.l.**[m] | 460–860 | 285–550 | 3–140 |

**Table S4|** Overview of the forest experimental plots examined, with the corresponding study site, coordinates and main tree type^2^.

| **Plot** | **Exploratory** | **latitude** | **longitude** | **Main tree type** |
| --- | --- | --- | --- | --- |
| AEW05 | Swabian Alb | 48.41961847 | 9.414681796 | Beech |
| AEW06 | Swabian Alb | 48.39405132 | 9.445936708 | Beech |
| AEW07 | Swabian Alb | 48.39623841 | 9.261355982 | Beech |
| AEW08 | Swabian Alb | 48.38258955 | 9.382384493 | Beech |
| AEW09 | Swabian Alb | 48.36934576 | 9.415217623 | Beech |
| AEW19 | Swabian Alb | 48.48424457 | 9.311183757 | Beech |
| AEW20 | Swabian Alb | 48.37010376 | 9.318719503 | Beech |
| AEW22 | Swabian Alb | 48.38230724 | 9.452638141 | Beech |
| AEW23 | Swabian Alb | 48.38583174 | 9.487975291 | Beech |
| AEW27 | Swabian Alb | 48.40023261 | 9.474351501 | Beech |
| AEW28 | Swabian Alb | 48.49235467 | 9.305009907 | Beech |
| AEW29 | Swabian Alb | 48.38366171 | 9.356606222 | Beech |
| AEW30 | Swabian Alb | 48.37273337 | 9.371377347 | Beech |
| AEW39 | Swabian Alb | 48.37578824 | 9.236548625 | Beech |
| AEW40 | Swabian Alb | 48.49960223 | 9.349436605 | Beech |
| AEW41 | Swabian Alb | 48.36441214 | 9.404138052 | Beech |
| AEW47 | Swabian Alb | 48.41670204 | 9.407903319 | Beech |
| AEW49 | Swabian Alb | 48.44536875 | 9.476394288 | Beech |
| AEW50 | Swabian Alb | 48.39211717 | 9.254448341 | Beech |
| HEW05 | Hainich-Dün | 51.26387813 | 10.24095787 | Beech |
| HEW06 | Hainich-Dün | 51.26771983 | 10.23937826 | Beech |
| HEW08 | Hainich-Dün | 51.35579218 | 10.51696741 | Beech |
| HEW09 | Hainich-Dün | 51.13024193 | 10.38114965 | Beech |
| HEW10 | Hainich-Dün | 51.08998735 | 10.46243387 | Beech |
| HEW12 | Hainich-Dün | 51.10068810 | 10.45518298 | Beech |
| HEW14 | Hainich-Dün | 51.36616394 | 10.25815169 | Beech |
| HEW15 | Hainich-Dün | 51.20507717 | 10.39016191 | Beech |
| HEW17 | Hainich-Dün | 51.28281358 | 10.22690385 | Beech |
| HEW21 | Hainich-Dün | 51.19427830 | 10.31900538 | Beech |
| HEW23 | Hainich-Dün | 51.27821774 | 10.20844805 | Beech |
| HEW24 | Hainich-Dün | 51.04650269 | 10.49121163 | Beech |
| HEW32 | Hainich-Dün | 51.13952828 | 10.35859032 | Beech |
| HEW34 | Hainich-Dün | 51.08471083 | 10.45427360 | Beech |
| HEW36 | Hainich-Dün | 51.10627577 | 10.40930317 | Beech |
| HEW38 | Hainich-Dün | 51.07935906 | 10.46017454 | Beech |
| HEW40 | Hainich-Dün | 51.07994428 | 10.49825888 | Beech |
| HEW41 | Hainich-Dün | 51.10805619 | 10.45493741 | Beech |
| HEW47 | Hainich-Dün | 51.17893474 | 10.37834387 | Beech |
| SEW05 | Schorfheide-Chorin | 53.05703356 | 13.88536623 | Beech |
| SEW06 | Schorfheide-Chorin | 52.90744279 | 13.84168805 | Beech |
| SEW07 | Schorfheide-Chorin | 53.10734802 | 13.69441893 | Beech |
| SEW08 | Schorfheide-Chorin | 53.19179716 | 13.93033786 | Beech |
| SEW35 | Schorfheide-Chorin | 52.91126795 | 13.85341878 | Beech |
| SEW36 | Schorfheide-Chorin | 52.95136417 | 13.75414255 | Beech |
| SEW37 | Schorfheide-Chorin | 52.94002157 | 13.78261215 | Beech |
| SEW38 | Schorfheide-Chorin | 52.88848023 | 13.67073695 | Beech |
| SEW39 | Schorfheide-Chorin | 52.92475573 | 13.86644547 | Beech |
| SEW40 | Schorfheide-Chorin | 52.91476876 | 13.86236515 | Beech |
| SEW41 | Schorfheide-Chorin | 52.91229384 | 13.90992535 | Beech |
| SEW42 | Schorfheide-Chorin | 52.90274751 | 13.91453434 | Beech |
| SEW43 | Schorfheide-Chorin | 52.90097652 | 13.92832556 | Beech |
| SEW44 | Schorfheide-Chorin | 52.91845902 | 13.85922060 | Beech |
| SEW45 | Schorfheide-Chorin | 53.04545452 | 13.83788884 | Beech |
| SEW46 | Schorfheide-Chorin | 53.07212245 | 13.77744397 | Beech |
| SEW47 | Schorfheide-Chorin | 53.07127806 | 13.77136543 | Beech |
| SEW49 | Schorfheide-Chorin | 52.88526043 | 13.89123063 | Beech |
| SEW50 | Schorfheide-Chorin | 53.03952843 | 13.78704314 | Beech |

Table S5| **Summary of normalization procedure.**

|  | smallest **root** sample: HEW06_root | smallest soil sample: SEW47_soil | aligned **soil** sample (normalization) |
| --- | --- | --- | --- |
| observed OTU | 64 | 204 | 169.63 |
| Chao1 estimation | 181.167 | 480.12 | 480.12 |
| standard error Chao1 | 80.68 | 81.54 | 81.54 |
| observed OTU [%] | 35.33 | 42.49 | 35.33 |
| Chao1 estimation [%] | 100 | 100 | 100 |
| number of sequences | **265** | 1945 | **1415** |

**Table S6| Summary of Procrustes-based comparisons of all 10 subsets without rare OTUs (>3 reads) generated via random selection of sequences after the normalization procedure.**

| Comparison: repetition subsample … vs … | Procrustes Sum of Squares | Correlation in a symmetric Procrustes rotation | Significance |
| --- | --- | --- | --- |
| 1 vs 2 | 0.01278 | 0.9936 | **0.001** |
| 1 vs 3 | 0.01082 | 0.9946 | **0.001** |
| 1 vs 4 | 0.01222 | 0.9939 | **0.001** |
| 1 vs 5 | 0.01173 | 0.9941 | **0.001** |
| 1 vs 6 | 0.01295 | 0.9935 | **0.001** |
| 1 vs 7 | 0.01268 | 0.9936 | **0.001** |
| 1 vs 8 | 0.01206 | 0.994 | **0.001** |
| 1 vs 9 | 0.01302 | 0.9935 | **0.001** |
| 1 vs 10 | 0.01079 | 0.9946 | **0.001** |
| 2 vs 3 | 0.01192 | 0.994 | **0.001** |
| 2 vs 4 | 0.01398 | 0.993 | **0.001** |
| 2 vs 5 | 0.01253 | 0.9937 | **0.001** |
| 2 vs 6 | 0.01196 | 0.994 | **0.001** |
| 2 vs 7 | 0.01249 | 0.9937 | **0.001** |
| 2 vs 8 | 0.01334 | 0.9933 | **0.001** |
| 2 vs 9 | 0.01725 | 0.9913 | **0.001** |
| 2 vs 10 | 0.01318 | 0.9934 | **0.001** |
| 3 vs 4 | 0.0123 | 0.9938 | **0.001** |
| 3 vs 5 | 0.01273 | 0.9936 | **0.001** |
| 3 vs 6 | 0.01254 | 0.9937 | **0.001** |
| 3 vs 7 | 0.01336 | 0.9933 | **0.001** |
| 3 vs 8 | 0.01084 | 0.9946 | **0.001** |
| 3 vs 9 | 0.0166 | 0.9917 | **0.001** |
| 3 vs 10 | 0.01091 | 0.9945 | **0.001** |
| 4 vs 5 | 0.01411 | 0.9929 | **0.001** |
| 4 vs 6 | 0.01362 | 0.9932 | **0.001** |
| 4 vs 7 | 0.01518 | 0.9924 | **0.001** |
| 4 vs 8 | 0.01242 | 0.9938 | **0.001** |
| 4 vs 9 | 0.01914 | 0.9904 | **0.001** |
| 4 vs 10 | 0.01168 | 0.9941 | **0.001** |
| 5 vs 6 | 0.01343 | 0.9933 | **0.001** |
| 5 vs 7 | 0.01251 | 0.9937 | **0.001** |
| 5 vs 8 | 0.01247 | 0.9937 | **0.001** |
| 5 vs 9 | 0.0152 | 0.9924 | **0.001** |
| 5 vs 10 | 0.01444 | 0.9928 | **0.001** |
| 6 vs 7 | 0.01132 | 0.9943 | **0.001** |
| 6 vs 8 | 0.01132 | 0.9943 | **0.001** |
| 6 vs 9 | 0.01044 | 0.9948 | **0.001** |
| 6 vs 10 | 0.0112 | 0.9944 | **0.001** |
| 7 vs 8 | 0.01314 | 0.9934 | **0.001** |
| 7 vs 9 | 0.01363 | 0.9932 | **0.001** |
| 7 vs 10 | 0.01232 | 0.9938 | **0.001** |
| 8 vs 9 | 0.01245 | 0.9938 | **0.001** |
| 8 vs 10 | 0.01106 | 0.9945 | **0.001** |
| 9 vs 10 | 0.01344 | 0.9933 | **0.001** |

Supplemental references

1. Schall, P. & Ammer, C. How to quantify forest management intensity in Central European forests. *Eur. J. For. Res.***132.2**, 379-396 (2013).

2. Fischer, M. *et al.* Implementing large-scale and long-term functional biodiversity research: The Biodiversity Exploratories. *Basic Appl. Ecol.* 11, 473-485 (2010).

3. Solly, E. F. *et al.* Factors controlling decomposition rates of fine root litter in temperate forests and grasslands. *Plant Soil* 382, 203-218 (2014).
